# Can flow cytometric measurements of reactive oxygen species levels determine minimal inhibitory concentrations and antibiotic susceptibility testing for Acinetobacter baumannii?

**DOI:** 10.1371/journal.pone.0305939

**Published:** 2024-06-24

**Authors:** Jia Hao Yeo, Jia Qian Low, Nasren Begam, Wan-Ting Leow, Andrea Lay-Hoon Kwa

**Affiliations:** 1 Department of Pharmacy, Singapore General Hospital, Singapore, Singapore; 2 SingHealth-Duke-NUS Academic Clinical Programme, Singapore, Singapore; 3 Department of Pharmacy, NUS, Singapore, Singapore; 4 Emerging Infection Diseases Program, Duke-NUS Graduate Medical School, Singapore, Singapore; Jahangirnagar University, BANGLADESH

## Abstract

Current antimicrobial susceptibility testing (AST) requires 16–24 hours, delaying initiation of appropriate antibiotics. Hence, there is a need for rapid AST. This study aims to develop and evaluate the feasibility of a rapid flow cytometric AST assay to determine minimum inhibitory concentration (MIC) for carbapenem-resistant *Acinetobacter baumannii* (CRAB). Antibiotic exposure causes increased intracellular reactive oxygen species (ROS) in bacteria. We hypothesized that ROS can be used as a marker to determine MIC. We assessed three CRAB clinical isolates across fifteen antibiotics at various concentrations in a customized 96-well microtiter plate. The antibiotics assessed include amikacin, beta-lactams (ampicillin/sulbactam, aztreonam, cefepime, ceftolozane/tazobactam, doripenem, imipenem, meropenem, and piperacillin/tazobactam), levofloxacin, polymyxin B, rifampicin, trimethoprim/sulfamethoxazole, and tetracyclines (tigecycline and minocycline). These clinical CRAB isolates were assessed for ROS after antibiotic treatment. Increased ROS levels indicated by increased RedoxSensor^TM^ Green (RSG) fluorescence intensity was assessed using flow cytometry (FCM). MIC was set as the lowest antibiotic concentration that gives a ≥1.5-fold increase in mode RSG fluorescence intensity (MIC_RSG_). Accuracy of MIC_RSG_ was determined by comparing against microtiter broth dilution method performed under CLSI guidelines. ROS was deemed accurate in determining the MICs for β-lactams (83.3% accuracy) and trimethoprim/sulfamethoxazole (100% accuracy). In contrast, ROS is less accurate in determining MICs for levofloxacin (33.3% accuracy), rifampicin (0% accuracy), amikacin (33.3% accuracy), and tetracyclines (33.3% accuracy). Collectively, this study described an FCM-AST assay to determine antibiotic susceptibility of CRAB isolates within 5 hours, reducing turnaround time up to 19 hours.

## Introduction

The *Acinetobacter baumannii* pathogen causes a range of life-threatening nosocomial infections [1]. The *A. baumannii* infections are associated with poor clinical outcomes, including attributable mortality rates of 8.4–36.5% [2] and excess 5–10 days of ICU stay [3,4]. Early treatment with appropriate antibiotics is vital to reduce mortality. Inappropriate antibiotics therapy can lead to increased risk of in-hospital mortality (adjusted relative risk ratio 1.76) [5] and increased 30-day mortality of 43.7% [6]. Delays in initiating appropriate antibiotics by ≥1 day is associated with 6.6% increase in mortality [7].

Appropriate treatment is further complicated by global increasing rates of resistance [8,9]. Multi-drug resistant (MDR) *A. baumannii* infection is associated with 5-fold increase in relative risk of receiving inappropriate empirical therapy [5]. Global investigation of multidrug-resistant A. baumannii revealed that resistance rates of imipenem (a carbapenem) increased exponentially every 5 years from 2000 to 2016 among countries in the Organization for Economic Cooperation and Development [10]. A study assessing Acinetobacter spp. collected from 140 hospitals across 32 countries revealed non-susceptibility to carbapenems doubled from 2005 to 2009 [11]. European surveillance data from 2009 showed that the Acinetobacter *spp*. was implicated in intensive care unit (ICU)-acquired pneumonia up to 21.8% of the time, in ICU-acquired bloodstream infections up to 17.1% and in ICU-acquired urinary tract infections up to 11.9% [12]. In Singapore, imipenem resistance rates in the largest tertiary acute care hospital, Singapore General Hospital in 2011–2015 was 4.3% higher compared to 2006–2008 [13]. Patients may only receive appropriate antibiotics after antimicrobial susceptibility testing (AST). Therefore, a short turn-around time to generate AST results is a crucial factor in ensuring early administration of appropriate antibiotics and successful treatment.

Current AST methods involve minimum inhibitory concentration (MIC) determination using culture-based methods, such as broth dilution and agar dilution assays. These culture methods are gold standards with high levels of sensitivity and specificity. However, current AST methods require 16–24 hours incubation following isolation of pure bacterial colonies [14,15]. This delays administration of appropriate antibiotics thereby increasing the likelihood of infection-related mortality. While rapid AST methods such as molecular and mass spectrometry-based methods were proposed and have been compared against the gold standards, these rapid AST methods may not always specify antibiotic susceptibility [16].

Flow cytometry (FCM) has been considered for rapid AST since the 1980s [17,18]. FCM can assess more than 10,000 cells per second. This rapid methodology will not only reduce the turnaround time significantly, delivering same-day results but is also able to detect bacteria sub-populations within the strains such as heteroresistant sub-populations. Currently, there is limited literature establishing a FCM assay that can be adopted for rapid AST covering a wide range of antibiotics [19–21]. Flow cytometry has been demonstrated to have good correlation to current automated diagnostic methods [22]. To the best of our knowledge, flow cytometric AST has yet to be established for routine clinical use globally.

Reactive oxygen species (ROS) is a collective term for all unstable, reactive oxygen-containing molecules. Exposing bacteria to antibiotics increases intracellular ROS, causing oxidative stress in bacteria [23–27]. Increased ROS can be simply measured and assessed by fluorometric methods such as flow cytometry. Therefore, we hypothesized that ROS is a suitable marker in predicting MIC and antibiotic susceptibility using FCM. Using ROS as an indicator, we aimed to evaluate the feasibility of an “one-protocol-fits-all-antibiotics” FCM-AST assay.

## Materials and methods

### Bacteria isolates

Nonclonal clinical strains of CRAB previously collected from the largest tertiary hospital in Singapore as part of a nationwide surveillance study from 2009 to 2016, were used in this study. Genus identity was determined using Vitek 2 ID-GN cards (bioMérieux, Inc., Hazelwood, MO). Bacteria isolates were stored at -80°C. Isolates were sub-cultured twice on Tripticase Soy Agar (TSA) with 5% sheep blood (Thermo Fisher, Singapore) at 35°C before each experiment. Isolates were randomly selected for this study. Three CRAB clinical isolates (AB0047, AB0356, AB0603) were used in this study (S1 Table in S1 File). The lab adapted ATCC19606 strain was used as a reference strain for characterizing the RSG fluorophore.

### Fluorophores used for FCM

The fluorescent dye, RedoxSensor^TM^ Green (RSG) of the *BacLight* RedoxSensor Green Vitality Kit (Thermo Fisher Scientific, Singapore), was used to assess bacterial ROS. Increased RSG fluorescence intensity indicates increased intracellular ROS. To identify viable bacteria for ROS assessments, the fluorescent dyes, SYTO-62 and propidium iodide (PI), (Thermo Fisher Scientific, Singapore) were used together with RSG. SYTO-62 labels nucleic acid of all bacteria. PI only enters non-viable bacteria with compromised membranes and intercalate DNA.

### Characterization of RSG fluorophore

The ATCC 19606 *A. baumannii* reference strain was used to characterize the RSG fluorophore. An inoculum of 10^5.7^ bacteria were stained with 20 μM RSG at room temperature for 15 mins. Stained bacteria were then exposed to various ROS-generating reagents. Hydrogen peroxide and sodium hypochlorite were added at a final concentration of 10 mM and 1.5 mM respectively. Hydroxyl radicals were generated using 1mM ferrous sulfate heptahydrate reacting with 200 μM hydrogen peroxide [28]. Samples were then assessed by FCM at every timepoint.

### FCM assessments of MIC

Similar to standard microtiter broth dilution method, bacteria suspension at 10^5.7^ CFU/mL was inoculated into each well of a customized microtiter broth dilution panel. The plate was incubated at 35°C for 30 minutes. Bacteria were then stained with the fluorescence cocktail (20 μM RSG, 1 μM SYTO-62 and 20 μM PI) and incubated for a further 15 minutes at room temperature before FCM assessments. Data acquisition was carried out with CytoFlex (Beckman Coulter, Singapore). Details on instrumental settings and configurations during data acquisition can be found in (S2 Table in S1 File). The manufacturer settings with a default “medium” flow rate of 30 μL/min was used. Each well was sampled for 180 seconds or until 20,000 events were collected, whichever came first. The durations for sample mixing and backflush of the fluidic system were set at 5 s and 60 s respectively to ensure accuracy of FCM assessments. Data acquired were exported in .fcs files and analyzed using the FlowJo software (v10.4, Tree Star, Ashland, OR, USA). Gating strategy and compensation matrix can be found in the (S1 Fig in S1 File and S2 Table in S1 File).

### Standard microtiter broth dilution method

MIC was determined using standard microtiter broth dilution method with customized 96-well microtiter broth dilution panels (Trek Diagnostics, East Grinstead, UK) performed under CLSI guidelines. Briefly, bacterial suspension of 10^7.7^ CFU/mL in physiological saline (0.85% (w/v) sodium chloride) was prepared. Ten-fold dilutions were performed to achieve 10^5.7^ CFU/mL in cation-adjusted Müller-Hinton broth. Microtiter broth dilution panel was inoculated with 100 μL of bacterial suspension at 10^5.7^ CFU/mL per well. Bacterial growth is assessed after the microtiter plate was then incubated at 35°C over 20–24 hours. Susceptibility breakpoints from MICs were interpreted primarily based on CLSI guidelines. Breakpoints from other sources were used when the breakpoint is not indicated in CLSI.

## Results

### Establishing optimal conditions for RedoxSensor Green to measure intracellular ROS

The commercial RedoxSensor™ Green (RSG) fluorophore can assess bacterial intracellular redox environment [29]. The RSG probe was used to sort dormant bacteria known as persisters, based on intracellular redox environment [30,31]. We first established optimal conditions for RSG to detect specific intracellular ROS.

**Fig 1:**
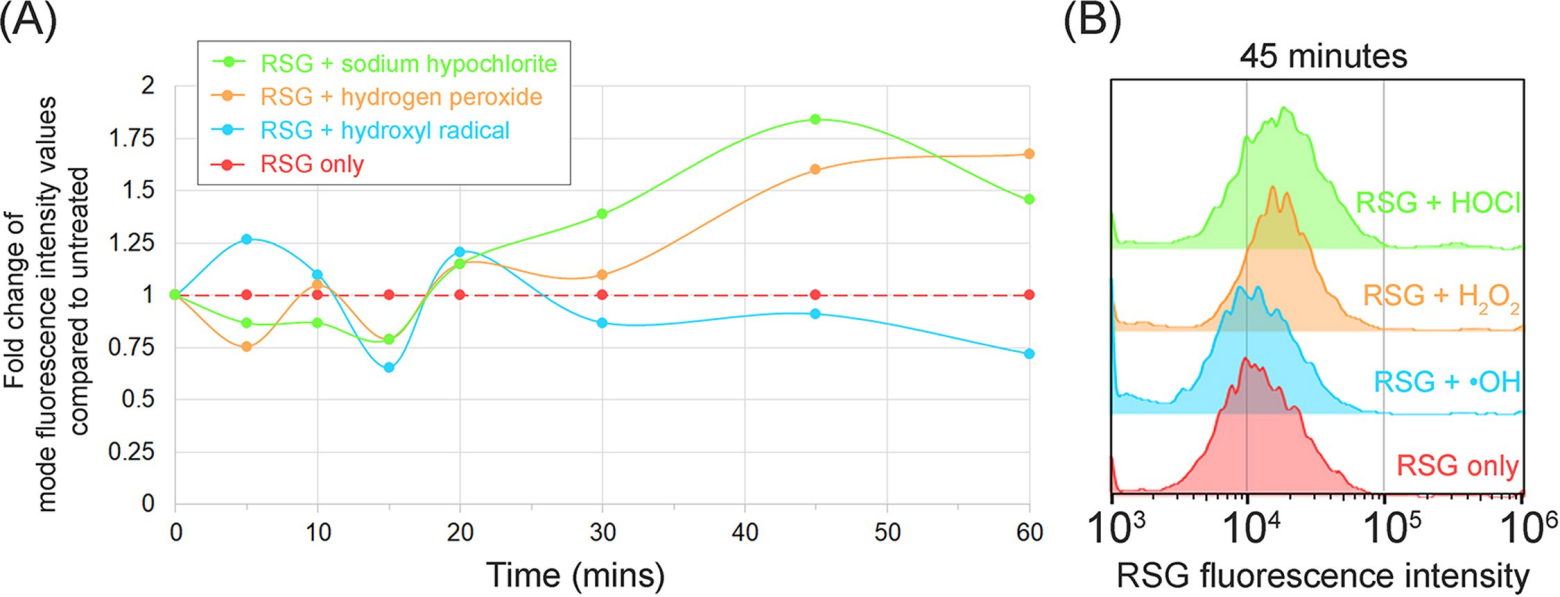
Establishing a FCM assay by characterising RSG for assessing oxidative stress in antibiotic exposed *A. baumannii*. The lab adapted strain, ATCC 19606, was first stained with RSG, before exposing to the ROS-generating reagents at room temperature. Bacteria were then assessed by FCM. Mode fluorescence intensities obtained from flow cytometric analyses were compared against untreated conditions as shown in panel (A). Flow cytometric histograms revealing the optimal fold change of RSG fluorescence intensities at 45 minutes were shown in panel (B).

To minimize bacterial redox buffering response to the ROS-generating reagents, the ATCC 19606 reference strain was first stained with RSG before exposing to ROS-generating reagents. Bacteria were then assessed using FCM. The RSG mode fluorescence intensities (peak of histogram) obtained from flow cytometric analyses were then compared against untreated conditions (**Fig 1; panel A**).

As recommended by the consensus established, we developed our FCM assay with an attempt to define the specific ROS measured by RSG [32]. Both hydrogen peroxide (H_2_O_2_) and hypochlorite (HOCl^-^) revealed an increase in RSG mode fluorescence intensity (MFI) compared to untreated bacteria at 45 minutes (**Fig 1; panel B**). Alterations in metabolism associated with oxidative damage are present as short as 30 minutes of antibiotics exposure. [33]. Therefore, in selecting a shorter antibiotic exposure time, we anticipate an increase in viability of bacteria for better ROS assessments. Hence, 45 minutes was used for FCM measurements of antibiotic-treated bacteria in this study. The ≥1.5-fold increase observed was also used as the minimum cut-off for increased ROS in subsequent experiments.

### Flow cytometric AST assay to predict MIC using ROS as a marker

**Fig 2:**
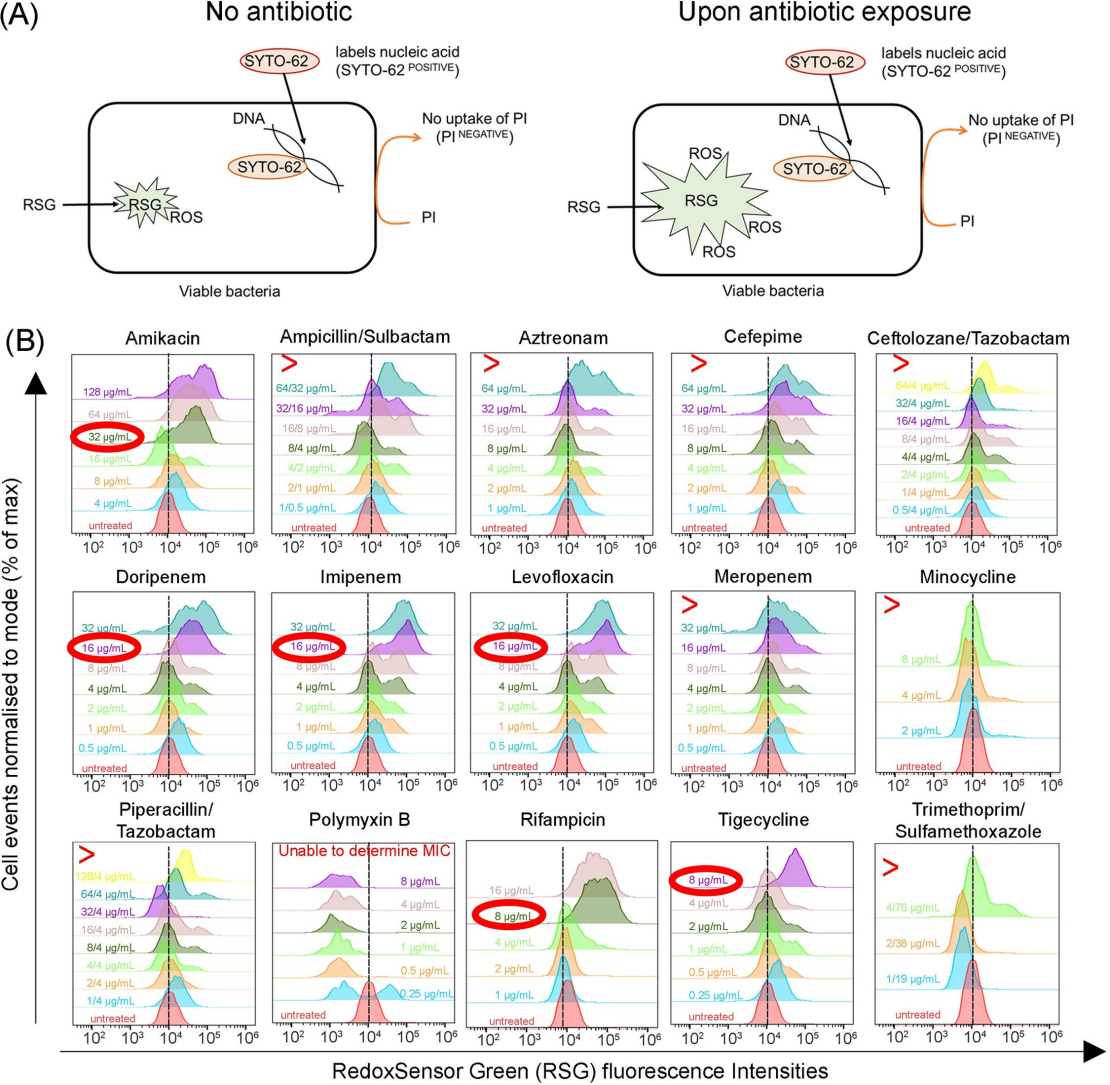
RSG fluorescence intensities in bacteria changes upon antibiotics exposure. Panel (A) describes the labelling strategy to assess ROS in viable bacteria using a cocktail of dyes. SYTO-62 labels all bacteria by binding to nucleic acid, while propidium iodide (PI) only enters non-viable bacteria. Viable bacteria (takes up SYTO-62 but not PI) will be assessed for ROS. Basal ROS is generated as a by-product of respiration under homeostatic conditions, hence exhibiting RSG fluorescence. After bacteria were exposed to antibiotics, there will be an increase in ROS. This increase in intracellular ROS, increases the intensity of RSG fluorescence measurable by flow cytometry. (B) Representative flow cytometric histograms depicting the changes in RSG fluorescence intensities in clinical isolate (AB0047) upon exposure to various antibiotics. Flow cytometric histograms for clinical isolates (AB0356 and AB0603) are shown in S2 and S3 Figs in S1 File. Exposure to sufficient concentrations of antibiotics resulted in increased RSG fluorescence as indicated by rightward shifts of the histograms. The antibiotic concentration corresponding to the first histogram shift compared to untreated (no antibiotics) was determined to be the MIC of that antibiotic (circled in red). The ‘>‘ symbol indicates MIC is higher than the highest concentration tested in the customized microtiter panel.

We next investigated ROS as a marker in our assay to predict MIC. Using a cocktail of various fluorophores, we assessed the oxidative stress in viable bacteria after exposure to antibiotics (**Fig 2; Panel A**). This fluorophore cocktail consists of RSG, SYTO-62 and propidium iodide (PI). Viable bacteria are defined as bacteria stained with SYTO-62 but not PI.

Bacteria strains were first exposed to varying concentrations of antibiotics in a customised microtiter panel for 30 minutes. These antibiotics include amikacin, ampicillin/sulbactam, aztreonam, cefepime, ceftolozane/tazobactam, doripenem, imipenem, levofloxacin, meropenem, minocycline, piperacillin/tazobactam, polymyxin-B, rifampicin, tigecycline and trimethoprim/sulfamethoxazole. Without removing the antibiotics, bacteria were then stained with the fluorescence cocktail for a further 15 minutes at room temperature. This staining protocol was adapted from protocol suggested by manufacturers, using metabolic inhibitors.

Flow cytometric analyses revealed increased RSG fluorescence intensities of viable bacteria populations after exposure to several antibiotics (**Fig 2; Panel B**). Increased RSG fluorescence intensities indicate increased ROS levels, supporting the basis that antibiotics exposure increases ROS production [23–27].

To avoid bias from human curation in determining MIC, we opted an arithmetic approach in determining MIC from the flow cytometry plots. Mode values of RSG fluorescence intensities (i.e., peak of histograms) at each drug concentration were compared against untreated controls (**Fig 2**). Adapting from our RSG characterization studies (**Fig 1**), a ≥1.5-fold increase in RSG fluorescence indicates an increase in bacterial ROS. Sub-inhibitory concentrations of antibiotics will result in negligible oxidative stress [34]. Therefore, the MIC was determined from the lowest antibiotic concentration that gives a ≥1.5-fold increase in mode RSG fluorescence intensity (MIC_RSG_).

A summary of these MICs determined from RSG fluorescence assessed by flow cytometry is reflected in **Table 1**. As the viable bacteria exposed to polymyxin-B remains very low (< 10%), we are unable to determine the MICs for polymyxin-B.

**Table 1:** Antibiotics susceptibility profiles of clinical AB isolates.

*MIC (mg/L)*	*AB0047*		*AB0356*		*AB0603*
*Antibiotics*	MIC_BMD_	MIC_RSG_	MIC_BMD_	MIC_RSG_	MIC_BMD_	MIC_RSG_
***AMK***	≥256 (R)	32 (I)	**≤ 4 (S)**	**≤ 4 (S)**	**≥256 (R)**	**≥256 (R)**
***ATM*** ^**1**^	**≥128 (R)**	**≥128 (R)**	≥128 (R)	64 (R)	64 (R)	≥128 (R)
***C/T*** ^**2**^	**≥128/4 (R)**	**≥128/4 (R)**	≥128/4 (R)	1/0.5 (S)	64/4 (R)	≥128/4 (R)
***DOR***	**32 (R)**	**32 (R)**	32 (R)	1 (S)	32 (R)	≥64 (R)
***FEP***	**≥128 (R)**	**≥128 (R)**	64 (R)	≥128 (R)	**≥128 (R)**	**≥128 (R)**
***IPM***	≥64 (R)	32 (R)	**16 (R)**	**32 (R)**	32 (R)	≥64 (R)
***LVX***	**32 (R)**	**16 (R)**	16 (R)	>32 (R)	32 (R)	≥64 (R)
***MER***	**≥64 (R)**	**≥64 (R)**	32 (R)	1 (S)	32 (R)	≥64 (R)
***MIN***	4 (S)	≥16 (R)	≤ 2 (S)	4 (S)	8 (I)	≥16 (R)
***PMB***	0.5 (I)	Unable to determine	0.5 (I)	Unable to determine	**0.5 (I)**	**0.5 (I)**
***RIF*** ^**3**^	**≥32 (R)**	**≥32 (R)**	4 (R)	≥32 (R)	4 (R)	≥32 (R)
***SAM***	32/16 (R)	≥128/64 (R)	32/16 (R)	1/0.5 (S)	**≥128/64 (R)**	**≥128/64 (R)**
***SXT***	**≥8/152 (R)**	**≥8/152 (R)**	**≥8/152 (R)**	**≥8/152 (R)**	**≥8/152 (R)**	**≥8/152 (R)**
***TGC*** ^**4**^	2 (S)	8 (R)	**1 (S)**	**0.5 (S)**	1 (S)	≥16 (R)
***TZP***	**≥256/4 (R)**	**≥256/4 (R)**	≥256/4 (R)	128/4 (R)	**≥256/4 (R)**	**≥256/4 (R)**
***Inoculum***	10^5.76^	10^5.83^	10^6.13^	10^5.85^	10^5.92^	10^6.01^

Table shows MICs of the three clinical CRAB isolates (AB0047, AB0356, AB0603). MICs obtained were interpreted based on CLSI guidelines, otherwise stated. Breakpoints against aztreonam, ceftolozane/tazobactam, rifampicin and tigecycline against AB were determined using breakpoints stated in literature. MICs for respective antibiotic as determined by both techniques, found within within 1-fold dilution are deemed as a “hit” and are indicated in **bold**. Abbreviations**:** MIC_BMD_: Minimal inhibitory concentrations determined from standard microtiter broth dilution; MIC_RSG_: Minimal inhibitory concentrations determined from RSG mode fluorescence intensity fold differences; (S): Susceptible; (I): Intermediate; (R): Resistant; AMK: Amikacin; ATM: Aztreonam; C/T: Ceftolozane/Tazobactam; DOR: Doripenem; FEP: Cefepime; IPM: Imipenem; LVX: Levofloxacin; MER: Meropenem; MIN: Minocycline; PMB: Polymyxin B; RIF: Rifampicin; SAM: Ampicillin/Sulbactam; SXT: Trimethoprim/Sulfamethoxazole; TGC: Tigecycline; TZP: Piperacillin/Tazobactam; ^1^Breakpoints based on EUCAST P. aeruginosa breakpoints; ^2^Breakpoints for AB determined as: Susceptible ≤ 2/4 μg/mL; Resistant: ≥ 4/4 μg/mL [35]; ^3^Breakpoints based on CLSI Staphylococcus breakpoints; ^4^Breakpoints based on FDA Enterobacteriaceae breakpoints.

### Comparison of the methods: FCM AST against standard microtiter broth dilution method

To determine the accuracy of our FCM AST assay, the MICs of antibiotics determined by FCM were compared against the standard microtiter broth dilution method (**Table 1**). MICs determined by FCM that are within ± 2-fold dilutions of the MIC determined by microtiter broth dilution was deemed as accurate.

Flow cytometric determination of MICs using ROS as a marker was deemed accurate for β-lactams (83.3% accuracy) and trimethoprim/sulfamethoxazole (100% accuracy) as compared to the standard microtiter broth dilution method. In contrast, FCM is less accurate in determining the MICs for levofloxacin (33.3% accuracy), rifampicin (0% accuracy), amikacin (33.3% accuracy), and tetracyclines (33.3% accuracy) when using ROS as a marker.

We then further verify if the MICs predicted by the 2 different methods affects the antibiotic susceptibilities breakpoints (**Tables 1 and 2**). The susceptibilities breakpoints (i.e., susceptible/intermediate/resistant) were primarily inferred from the CLSI guidelines. The EUCAST or recommended breakpoints by literature [35] were used when susceptibility breakpoints were not available on CLSI. We observed that the susceptibilities profile remains >95% accurate for ß-Lactams, levofloxacin, rifampicin, and trimethoprim/sulfamethoxazole. In other words, the MICs from the two techniques were still giving similar antibiotic susceptibility breakpoints for ß-Lactams, levofloxacin, rifampicin, and trimethoprim/sulfamethoxazole. The susceptibilities profile for amikacin (33.3% accuracy) and the tetracyclines (16.7% accuracy) were vastly different when inferring from MICs of the two techniques. This implies that the antibiotic susceptibilities inferred from the MICs of the two techniques were different for amikacin and tetracyclines

**Table 2:** Summary of MICs comparisons between standard microtiter broth dilution method and MICs determined via FCM (using ROS as a marker).

Antibiotics	MICs within ± 2-fold dilution	Matched antibiotic susceptibility breakpoints
Amikacin	1/3 (33.3%)	1/3 (33.3%)
β-lactams	20/24 (83.3%)	23/24 (95.8%)
Levofloxacin	1/3 (33.3%)	3/3 (100%)
Rifampicin	0%	3/3 (33.3%)
Tetracyclines	2/6 (33.3%)	1/6 (33.3%)
Trimethoprim/sulfamethoxazole	3/3 (100%)	3/3 (100%)
Polymyxin B ^#^	1/1 (100%)	1/1 (100%)

Antibiotics of which the MICs are compared between the FCM and standard microtiter broth dilution method as grouped and shown in the first column. MICs determined from FCM analyses that falls within ±2-fold dilution of the MICs from standard microtiter broth dilution method were indicated as accurate in the second column. MICs predicted from both methods were interpreted using CLSI guidelines and the breakpoints were compared. Matched breakpoints were indicated as accurate and shown in the last column. ^#^Unable to determine MIC of polymyxin B for AB0047 and AB0356 strains due to very low viable cells (<10%) available for flow cytometric assessments of ROS.

## Discussion

The *A*.*baumannii* is an aerobic pathogen that causes a range of life-threatening nosocomial infections with poor clinical outcomes [1]. Early treatment with appropriate antibiotics is vital to reduce mortality. Thus, time taken to produce AST results is a crucial factor in ensuring early administration of appropriate antibiotics and successful treatment.

One major advantage of FCM is the rapid assessments of cells at the single cell resolution. FCM assay also offers an advantage in higher flexibility compared to current automated AST tools, such as Vitek-2 [22,36]. High correlations (>90%) of flow cytometric determination of AST against Vitek-2 was documented across multiple species [22]. These flexibilities include testing any bacteria species against any antibiotics, including antibiotics combinations [37–39].

A major pitfall of FCM-based assays for routine use is data analyses. FCM data analyses are often time-consuming, requiring high technical expertise and can be subjective with high user variability. To improve reproducibility and reduce user variability, we adopted auto-contouring gating (S2 Fig in S1 File). We envisioned an automated workflow from the extractions of mode fluorescence intensities and subsequent comparison to a single MIC value output without human intervention [40].

Fluorescence methodologies, such as FCM, remain one of the simplest measurements of ROS in live cells. ROS arises from basal metabolism as a by-product of electron transport chain [25]. Exposing bacteria to antibiotic activates stress response pathways leading to a higher increase of intracellular ROS [23–27]. In *A. baumannii*, polymyxins increase hydroxyl radicals [41], which can be further amplified with rifampicin [42]. Ajiboye and colleagues demonstrated increased superoxide ion generation, NAD^+^/NADH ratio and ADP/ATP ratio in *A. baumannii* when exposed to various antibiotics at 4X higher MIC [43–47]. Dwyer and colleagues demonstrated higher hydrogen peroxide (H_2_O_2_) levels with ampicillin exposure compared to norfloxacin, while superoxide (•O_2_^–^) levels remain similar for both antibiotics [23]. This suggests that specific ROS might be generated in bacteria, in response to different antibiotics. However, we are unable to discern the specific ROS for each antibiotic through our assay.

We reasoned that the inaccuracies in our assay stemmed from the different mechanisms of actions of individual antibiotics. Aminoglycosides are broad-spectrum antibiotics that inhibit protein synthesis, by primarily binding to the aminoacyl site of 16S ribosomal RNA within the 30S ribosomal subunit. Similar to our observation for amikacin in clinical isolates, multiple aminoglycosides at concentrations higher than MICs did not generate ROS in lab-adapted *A. baumannii* strains [48]. Polymyxin B binds and neutralizes the lipopolysaccharide of Gram-negative bacteria, disrupting membrane integrity. Due to the pharmacokinetics of polymyxin-B, 45 minutes might not be the optimal duration to assess viable cells for polymyxin-B induced oxidative stress. Decreased oxidative respiration were reported in *Escherichia coli* and *Staphylococcus aureus* treated with “bacteriostatic” antibiotics, such as rifampicin that inhibits bacterial RNA polymerase, tetracyclines that inhibit protein synthesis via inhibition of 30s ribosome and levofloxacin that inhibit DNA synthesis [49]. Consistent to our data, these antibiotics do not reveal an increase in RSG fluorescence intensity.

Literature had debated if ROS is the key mediator of antibiotic bactericidal effect [34,50,51]. This current study neither contests nor concurs with the association of ROS to be the cause of bactericidal effects of antibiotics. This study also does not dissect the mechanisms of antibiotics actions nor the bacterial redox defense mechanisms against antibiotics. Rather, this study builds on the foundation that by exposing bacteria to a stress stimulus, such as sufficient concentrations of antibiotics, ROS will be increased in bacteria causing oxidative stress.

We envisaged that measuring bacterial ROS can also be further used to detect heteroresistant sub-populations routinely. There is increasing evidence that heteroresistance can lead to treatment failure and detecting heteroresistance is crucial for appropriate antibiotics for a successful treatment outcome. [52,53]. Current gold-standard in determining heteroresistance is population analysis profiling, which is tedious and laborious for routine clinical laboratory diagnosis [54–56]. Single-cell assessments by FCM can detect heterogenous sub-populations rapidly. Isogenic resistant sub-populations have lower oxidative burst than the susceptible counterparts upon antibiotic exposure [57]. Hence, our protocol will reveal a multi-modal histogram when assessing RSG fluorescence.

Further studies will aim to optimize this setup in attaining our aim for an “one-protocol-fits-all-antibiotics” FCM-AST workflow for *A. baumannii*. Nevertheless, this current protocol still demonstrates that ROS is a good indicator for determining MIC for β-lactams and Trimethoprim/sulfamethoxazole.

In summary, this study described the workflow of a rapid FCM-AST assay to determine antibiotic susceptibility of CRAB isolates against fifteen different antibiotics. We demonstrate that ROS is a good indicator for determining MICs for β-lactams and Trimethoprim/sulfamethoxazole. The workflow can produce results within 5 hours, up to 19 hours earlier compared to standard AST methods. Our assay can potentially translate to faster initiation of appropriate antibiotics, and hence, improved clinical outcomes.

## Supporting information

S1 File All supplementary tables and figures can be found in the supporting information file “Yeo et al Supporting information.”. (DOCX)
