# Supplementary material for: Can flow cytometric measurements of reactive oxygen species levels determine minimal inhibitory concentrations and antibiotic susceptibility testing for Acinetobacter baumannii?
Source: PLoS One. 2024 Jun 24;19(6):e0305939. doi: 10.1371/journal.pone.0305939 (PMC11195951; doi:10.1371/journal.pone.0305939)
Supplement: S1 File — (DOCX) [file pone.0305939.s001.docx]

**SUPPLEMENTARY INFORMATION**

**1. Materials and methods**

**A. Isolates**

Nonclonal clinical strains of CRAB previously collected from the largest tertiary hospital in Singapore as part of a nationwide surveillance study from 2009 to 2016, were used in this study. Three CRAB clinical isolates (AB0047, AB0356, AB0603) were used in this study. More information of these isolates used in this study can be found in **S1 Table**.

**S1 Table: Features of the clinical *A. baumannii* strains used in this work.**

|  | AB0047 | AB0356 | AB0603 |
| --- | --- | --- | --- |
| Site | Wound | Urinary | Respiratory |
| Serotype | 1 | 491 | 1816 |
| Resistance genes | | | |
| ADC-10 | - | + | - |
| ADC-11 | - | + | - |
| ADC-73 | - | - | + |
| ADC-117 | + | - | - |
| OXA-23 | + | + | + |
| OXA-66 (OXA-51 subgroup) | - | - | + |
| OXA-69 (OXA-51 subgroup) | + | + | - |
| APH(3”)-Ia | + | - | - |
| APH(3'')-Ib | - | - | + |
| APH(3')-VIa | + | - | - |
| APH(6)-Id | - | - | + |
| *aadA* | + | - | - |
| AAC(3)-Ia | + | - | - |
| MphE | - | - | + |
| AmvA | + | + | + |
| AbaQ | + | + | + |
| AbaF | + | + | + |
| Tet(A) | + | - | - |
| Tet(B) | - | - | + |
| Tet(R) | - | - | + |
| *qacEdelta1* | + | - | - |
| AdeABC | + | + | + |
| AdeFGH | + | + | + |
| AdeIJK | + | + | + |
| AbeS | + | + | + |
| AbeM | + | + | + |
| *lpsB* | + | + | + |
| *gyrA* | + | + | + |
| *parC* | + | + | + |
| *armA* | - | - | + |
| *msrE* | - | - | + |
| *sul1* | + | - | - |

The “+” symbol in the table indicates presence of genes, while the “-“ symbol indicates absence of respective genes.

**B. Flow cytometry**

**I. Flow cytometer configuration**

A Cytoflex® flow cytometer (Beckman Coulter, Brea, CA, USA) at the basic 4+3+2 configuration was used in this study. The flow cytometer is equipped with 405 nm, 488 nm and 640 nm lasers to excite fluorophores. A summary of fluorophores detected by corresponding detectors and gain voltages applied to the respective detectors were shown in S2 Table.

**S2 Table: instrumental settings on the CytoFlex flow cytometer**

| **Parameter** | **Assessments/Purpose** | **Detector Gain** |
| --- | --- | --- |
| Forward Scattering (FSC)-H | Size | 800 V |
| Side Scattering (SSC)-H | Granularity | 2800 V |
| FITC-H | RedoxSensor Green | 800 V |
| PC5.5-H | Propidium iodide | 150 V |
| APC-H | SYTO-62 | 800 V |
| PB450-H  (empty fluorescence channel 1) | Noise elimination | 51 V (default) |
| KO525-H (empty fluorescence channel 2) | Noise elimination | 27 V (default) |

**II. Sample acquisition settings**

Flow cytometric data were acquired using the complementing CytExpert software (version 2.5). A manual threshold on forward scattering (height) and side scattering (height) was established at 10,000 and 20,000 respectively. Flow rates were set as manufacturer default for <medium> setting, which is denoted as 30 µL/min. Durations for sample mixing and backflush of the fluidics was set as 5 s and 60 s respectively to increase accuracy. Each well of the microtiter plate was sampled for 180 seconds or until 20,000 events were collected, whichever came first. A separate 96-wells microtiter plate, containing single stained controls, was prepared for compensating spectra overlap during analyses on the FlowJo software.

**III. Data analyses**

Acquired flow cytometric data were analysed using FlowJo (version 10.6; Treestar ® FlowJo, LLC, Ashland, OR, USA). Compensation against spectral overlap was also performed on the FlowJo software. Compensation matrix for spectral spillover is tabulated in S3 Table. Gating strategy used for identifying viable cells were shown in S1 Figure. Flow cytometric data were presented as overlaid or half-staggered histograms throughout manuscript for simplicity.


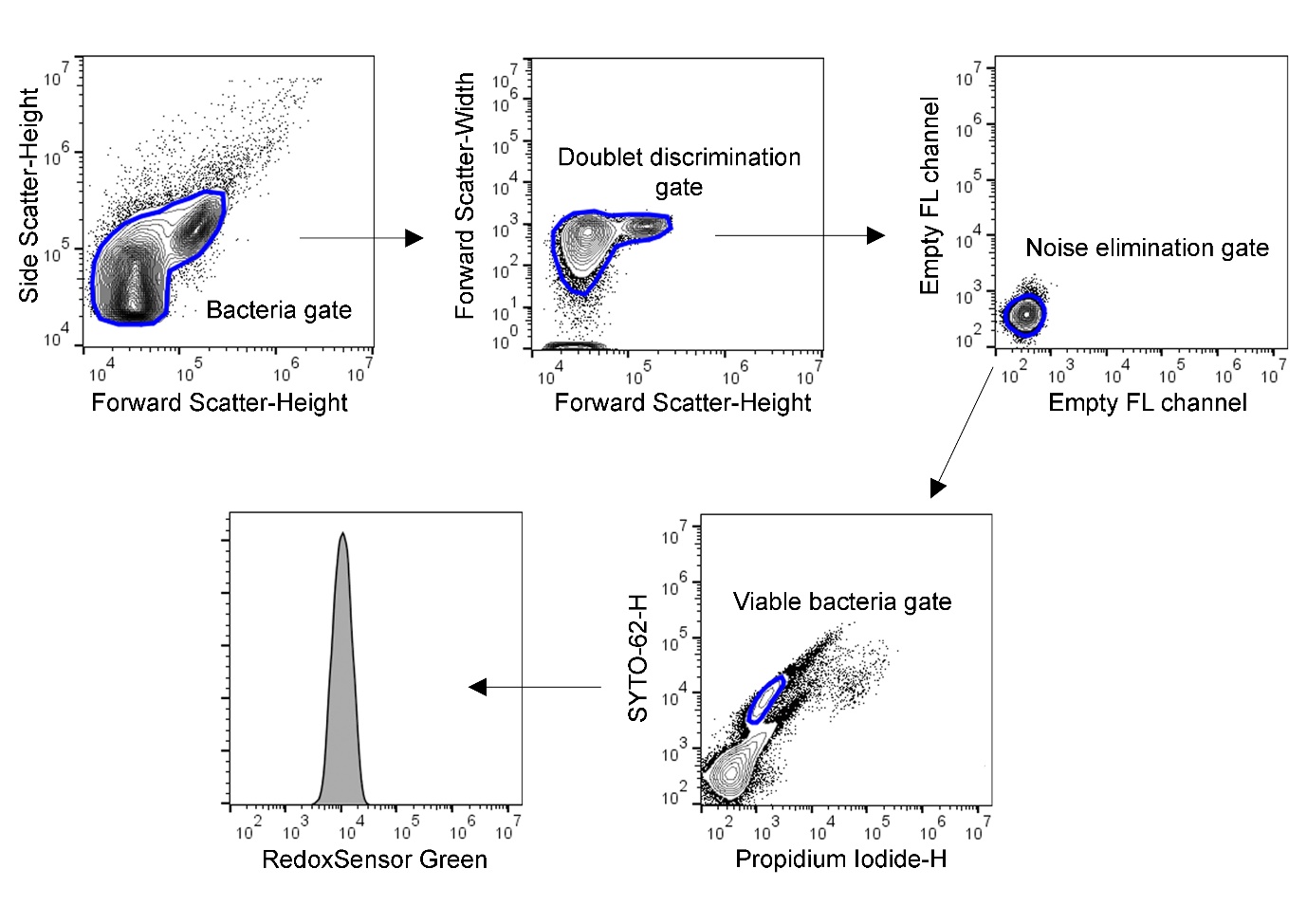

**S1 Figure:** Gating strategy used for analysing flow cytometry data to assess redox status in viable bacteria

*Schematic showing standardised gating strategy applied in flow cytometric data analyses. Gating identifies population of interest by placing a boundary (also known as gates) on flow cytometry plots. We implemented a gating strategy to identify redox status in viable bacteria. To minimize user-bias during flow cytometric analyses, the auto-contouring gate function on FlowJo analyses software was applied on contour plots at 2% threshold. These automated gates were placed to encompass as many concentric rings of contour as possible. A* ***bacteria gate*** *was first applied to identify bacteria using forward scatters (height) and side scatters (height). This is followed by a* ***doublet discrimination gate*** *to ensure only single cells are included for data analyses. Subsequently, a* ***noise elimination gate*** *was applied to reduce electronic noise for better accuracy and specificity. A* ***viable bacteria gate*** *was then placed for events that are SYTO-62^POSITIVE^Propidium-Iodide^NEGATIVE^. The viable bacteria gate is placed in reference to single stain controls to determine if populations are negative or positive for a marker/stain. Viable bacteria were then assessed for RSG fluorescence intensity (histogram). Histograms were then overlaid in a staggered manner, as presented throughout the manuscript.*

**S3 Table:** Compensation matrix used for spectral spill-over of each fluorophore into respective detector used for FCM.

| **Fluorophore\Detector** | **FL1-H: RSG** | **FL3-H: PI** | **FL5-H: SYTO-62** |
| --- | --- | --- | --- |
| **RSG** | 100 | 0.4 | 0 |
| **PI** | 0.5 | 100 | 19.5 |
| **SYTO-62** | 0 | 9.5 | 100 |

**2. RESULTS**

Flow cytometric plots for determining MICs for both isolates, AB0356 and AB0603, are shown in **Supplementary Figure-S2** and **Supplementary Figure-S3** respectively. Bacteria were first exposed to antibiotics before staining with fluorophores. Viable bacteria were then assessed using flow cytometry for redox status using RSG. Increase in ROS will be indicated by an increase in RSG fluorescence intensity, represented by a rightward shift of histogram compared to untreated.


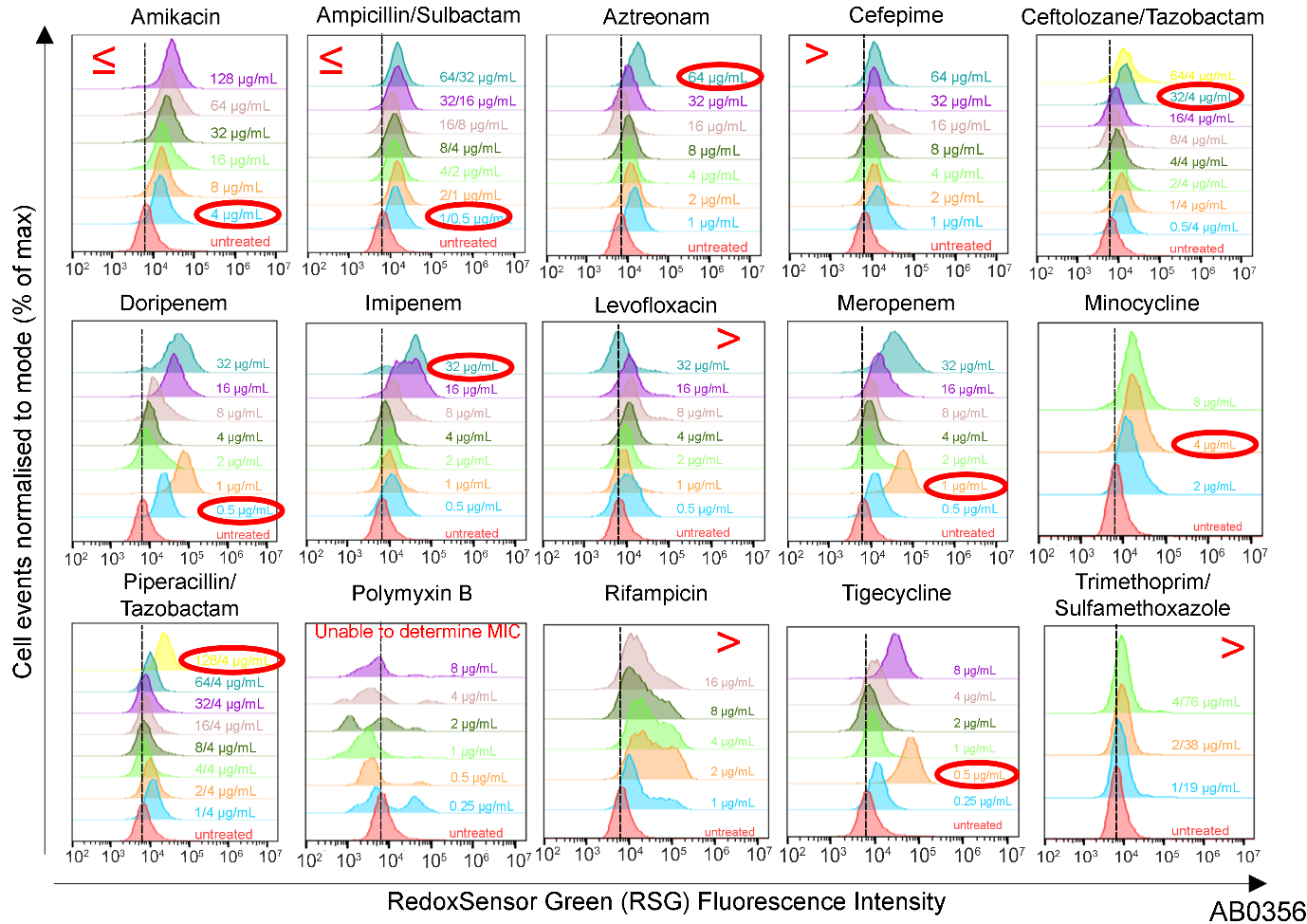


**S2 Figure**: Representative flow cytometry plots for determining MICs of antibiotics for AB0356

Representative flow cytometric histograms depicting the changes in RSG fluorescence intensities in clinical isolate (AB0356) after exposing to various antibiotics. Exposing bacteria to sufficient concentrations of antibiotics resulted in increased RSG fluorescence as indicated by rightward shifts of the histograms. The lowest antibiotic concentration corresponding to the first histogram shift compared to untreated (no antibiotics) was determined to be the MIC of that antibiotic (circled in red). The ‘>’ symbol indicates the MIC is higher than the highest concentration tested for that antibiotic in the customized microtiter panel.


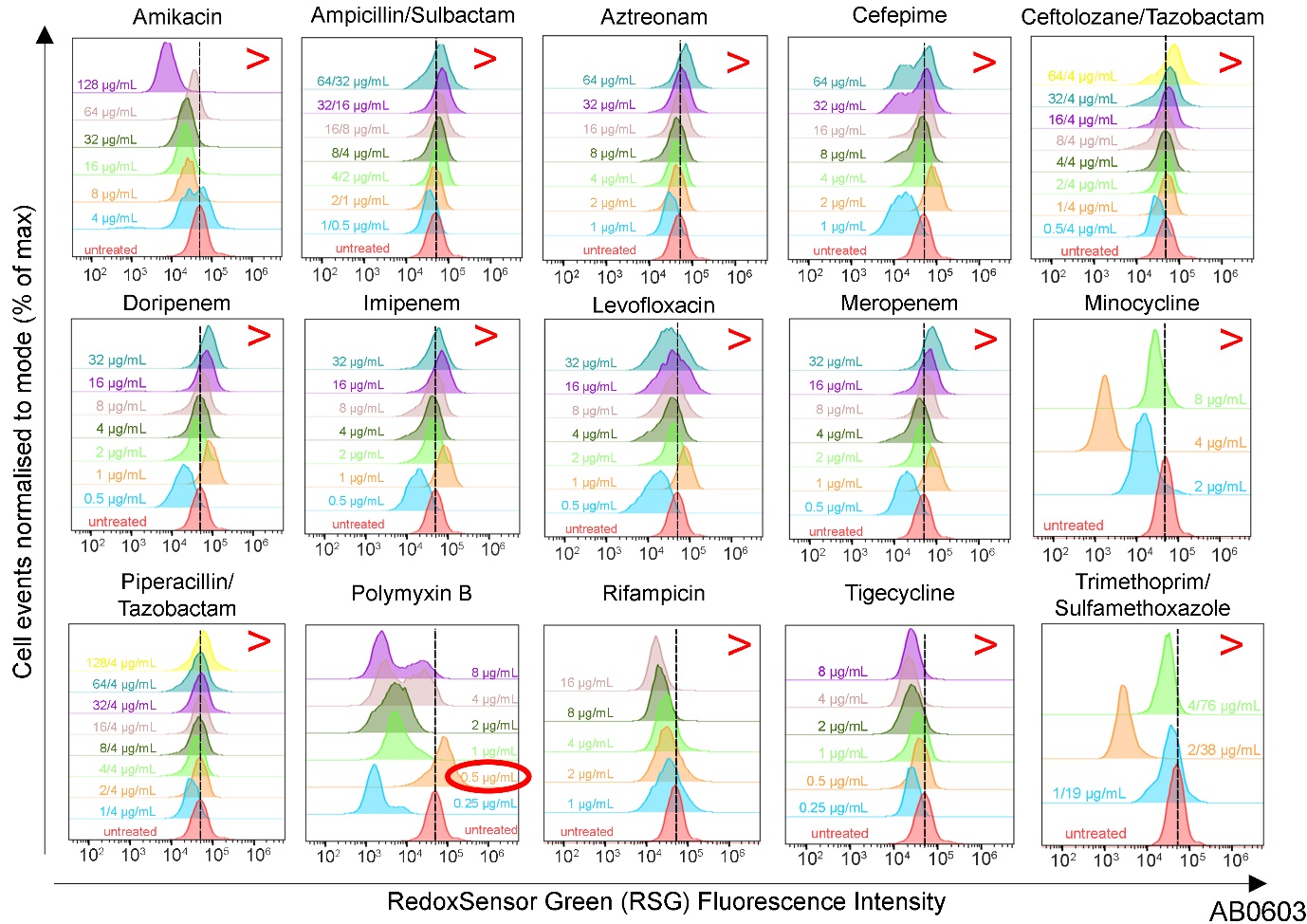


**S3 Figure**: Representative flow cytometry plots for determining MICs of antibiotics for AB0603

Representative flow cytometric histograms depicting the changes in RSG fluorescence intensities in clinical isolate (AB0356) upon exposure to various antibiotics. Exposing bacteria to sufficient concentrations of antibiotics resulted in increased RSG fluorescence as indicated by rightward shifts of the histograms. The lowest antibiotic concentration corresponding to the first histogram shift compared to untreated (no antibiotics) was determined to be the MIC of that antibiotic (circled in red). The ‘>’ symbol indicates MIC is higher than the highest concentration tested of that antibiotic in the customized microtiter panel.
